# Factors associated with the closure of obstetric units in German hospitals and its effects on accessibility

**DOI:** 10.1186/s12913-023-09204-1

**Published:** 2023-04-05

**Authors:** Jan Hoffmann, Till Dresbach, Carsten Hagenbeck, Nadine Scholten

**Affiliations:** 1 Faculty of Medicine and University Hospital Cologne, Faculty of Human Sciences, Institute for Medical Sociology, Health Services Research, and Rehabilitation Science (IMVR), University of Cologne, Eupener Str. 129, 50933 Cologne, Germany; 2 University Hospital Bonn, Department of Neonatology and Pediatric Intensive Care Medicine, Venusberg-Campus 1, 53127 Bonn, Germany; 3 Department of Obstetrics and Gynecology, University Hospital Düsseldorf, Moorenstraße 5, 40225 Düsseldorf, Germany

**Keywords:** Regionalization, Obstetrics, Accessibility, Germany, Closure, Centralization

## Abstract

**Background:**

An increase in regionalization of obstetric services is being observed worldwide. This study investigated factors associated with the closure of obstetric units in hospitals in Germany and aimed to examine the effect of obstetric unit closure on accessibility of obstetric care.

**Methods:**

Secondary data of all German hospital sites with an obstetrics department were analyzed for 2014 and 2019. Backward stepwise regression was performed to identify factors associated with obstetrics department closure. Subsequently, the driving times to a hospital site with an obstetrics department were mapped, and different scenarios resulting from further regionalization were modelled.

**Results:**

Of 747 hospital sites with an obstetrics department in 2014, 85 obstetrics departments closed down by 2019. The annual number of live births in a hospital site (OR = 0.995; 95% CI = 0.993–0.996), the minimal travel time between two hospital sites with an obstetrics department (OR = 0.95; 95% CI = 0.915–0.985), the availability of a pediatrics department (OR = 0.357; 95% CI = 0.126–0.863), and population density (low vs. medium OR = 0.24; 95% CI = 0.09–0.648, low vs. high OR = 0.251; 95% CI = 0.077–0.822) were observed to be factors significantly associated with the closure of obstetrics departments. Areas in which driving times to the next hospital site with an obstetrics department exceeded the 30 and 40 min threshold slightly increased from 2014 to 2019. Scenarios in which only hospital sites with a pediatrics department or hospital sites with an annual birth volume of ≥ 600 were considered resulted in large areas in which the driving times would exceed the 30 and 40 min threshold.

**Conclusion:**

Close distances between hospital sites and the absence of a pediatrics department at the hospital site associate with the closure of obstetrics departments. Despite the closures, good accessibility is maintained for most areas in Germany. Although regionalization may ensure high-quality care and efficiency, further regionalization in obstetrics will have an impact on accessibility.

## Background

Regionalization in the health care sector can be defined as “the development of a structured system of care to improve patient outcome by directing patients to facilities with optimal capabilities for a given type of illness or injury. The development of a regionalized system is typically driven by economic factors, such as the infeasibility of all hospitals to maintain the equipment and personnel to treat specific medical conditions, or by interhospital variations in patient outcomes within a geographic region” [1]. This definition of regionalization can also be applied to the development in perinatal care, a dyad of obstetric and neonatal care. In perinatal care, the idea of regionalization origins in the provision of high-quality specialized health care for sick neonates and children in the field of neonatal intensive care and pediatric trauma care [1]. Numerous studies show improved patient outcomes when perinatal care is regionalized and delivered in medical centers compared to smaller hospitals [2–8]. Evidence shows, that mostly high-risk pregnancies and births benefit from regionalization in perinatal care (decreased mortalities for deliveries in high-volume and high-level hospitals [9–12]), whereas mixed results on patient outcomes exist for low-risk pregnancies and births [13]. Nevertheless, internationally, the development in obstetrics, as one part of perinatal care, indicates a progressive regionalization and consolidation [14–19].

Consolidation of medical services in obstetrics often results in the closure of obstetrics departments. Causes cited in the literature for department closures include: Number of births, hospital ownership, teaching status, geographic location, and market density. Hung et al. for example state that the closure of rural obstetric units is significantly associated with low birth volume and private ownership [20]. Albert et al. highlighted that hospitals with birth numbers below 500 births a year are particularly prone to close their obstetrics department [13]. Further, in their analysis, Mennicken et al. suggest that especially obstetrics departments with low case numbers face financial struggles and conclude that on average, small obstetrics departments are more likely to make losses [21], whereas Croft observed that, in Philadelphia, only obstetric facilities that belonged to non-academic medical centers closed [22]. Combier et al. stated that in France obstetric departments especially closed down in rural areas [23]. Further, the distance to the next hospital offering the same services represents a competition factor in the hospital market. Competition on the one hand may increase quality of care[24, 25] but on the other hand may push competing hospitals in the same catchment area out of the market [26]. If hospital sites in the same catchment area offer the same services, patients have the choice and hospitals are at higher risks to lose patients to their competitors [27].

On the other hand, there is the demand for care close to home for obstetric services. In Germany, the Federal Joint Committee (G-BA)—the main resolution body in the health care system— determined that for patients requiring emergency care in the field of internal medicine and surgery, driving times of 30 min by car should not be exceeded. They further state that for patients requiring treatment in an obstetrics department driving times should not exceed 40 min by car, arguing that high-quality care (i.e., availability of a pediatrics department) is more important than short driving times [28]. Simultaneously, a maximum travel time of 30 min is considered necessary to reach obstetric services [15, 21, 29], arguing that instances such as peri-partum bleeding, hypertensive crisis, preeclampsia, eclamptic seizure, onset of preterm birth, premature rupture of membranes or uterine rupture require fast medical treatment [29–31]. Combier and colleagues stated that in France a travel times of 30 min or more to an obstetric facility were associated with negative patient outcomes (i.e. fetal heart rate anomalies or out-of-hospital births) [23].

In Germany, choice of hospital is not limited by health insurance. Hospital treatment is reimbursed on the basis of per-case rates (diagnosis related groups, DRGs), which are consistent throughout Germany. In addition, there are no regional differences in remuneration of births in Germany. Births at home or in birth centers are rare in Germany and occurred in only 1.57% of cases in 2019 [32].

This study aimed to determine how organizational factors (ownership, academic teaching status, annual number of live births), regional factors (population density, fertility rate), competitive factors (minimal travel time between two hospital sites with an obstetrics department), and quality factors (the availability of a pediatrics department) are associated with the closure of obstetrics departments in Germany. Subsequently, this study sought to determine the differences in accessibility of hospital sites with an obstetrics department for 2014 and 2019 to examine (i) which areas exceeded driving times of 30 or 40 min and (ii) how further regionalization impacts accessibility.

## Methods

### Data sources

#### Quality reports of German hospitals

For this analysis, we used secondary data from the structured quality reports of all acute hospital sites in Germany for the reporting years 2014 and 2019. The regulations of the G-BA obligate every hospital site to prepare and submit a quality report every year [19]. The quality reports contain information and key figures for individual hospital sites in Germany, such as the address, ownership of the hospital site, case numbers, operation and procedure codes (OPS), International Statistical Classification of Diseases and Related Health Problems (ICD), and information on what specialty departments are available at each hospital site. Our analyses are based on the data of all acute care hospital sites that published a structured quality report in 2014 and 2019. Day care hospitals and rehabilitation clinics were excluded since they do not offer obstetric services.

#### Population data

To determine the population density in the area of each hospital site, we used data from the municipal directory of cities in Germany by area, population, and population density for 2014 and 2019 provided by the German Federal Statistical Office [33].

#### Data on fertility rate

To determine the fertility rate in the area of each hospital site, we used data from the Federal Institute for Research on Building, Urban Affairs and Spatial Development in Germany [34].

### Data procurement

#### Quality reports of German hospitals

The structured quality reports of the hospital sites are freely available from the websites of the G-BA and the hospitals.

#### Fertility rate

The data on fertility rate provided by the Federal Institute for Research on Building, Urban Affairs and Spatial Development are freely available at www.inkar.de.

### Data operationalization

We operationalized the following variables for logistic regression: *obstetrics department closed by 2019, annual number of live births in hospitals*, *ownership*, availability of a *pediatrics department on site*, *academic teaching hospital*, population density, *fertility rate*, and *minimal travel time between two hospital sites with an obstetrics department.*

The *annual number of live births* at each hospital site were identified using the subsection of the OPS code 9–26 (measures accompanying birth): 9-262, 9-262.0, 9-262.1, 9-262.x, 9-262.y. Hospital sites were identified as hospitals with a pediatrics department if the quality reports of the respective hospital contained a specialist department of pediatrics.

Hospital departments were identified using the specialist department codes in the quality reports. We defined hospital sites with an obstetrics department as hospital sites with a department of obstetrics and/or gynecology. In addition, the hospital site was required to have documented live births in the reporting year according to OPS or ICD classification to be defined as a hospital with an obstetrics department. The variable *obstetrics department closed by 2019* was created for the 2014 dataset to indicate whether the department of obstetrics still existed in the 2019 quality reports. For the descriptive presentation of hospital sites for 2019, we included all hospital sites in our analyses that provided a quality report for that year and fulfilled the criteria of a department of obstetrics.

The variable *academic teaching hospital* was operationalized from information provided within the quality reports. The variable included the values “yes,” “no,” and “university hospital.”

Data on population density was linked to hospital data via the postal code provided in the quality reports. We followed the classification of degrees of urbanization by the Federal Statistical Office and categorized population density as low population density (≤ 100 inhabitants per km^2^), medium population density (> 100 and ≤ 500 inhabitants per km^2^), and high population density (> 1000 inhabitants per km^2^). Data on fertility rate was linked to hospital data via the postal code provided in the quality reports. We categorized fertility rate in low (≤ 1.3), medium (> 1.3 to ≤ 1.6), and high (> 1.6) for the presentation of descriptive results. Finally, a variable indicating the shortest travel time from one hospital site with an obstetrics department to the next hospital site with an obstetrics department was created. Values for this variable were established via the open-source routing for shortest paths in road networks using the R package OSRM (osrm package [3.4.1]).

### Data analysis

We performed multivariate logistic regression for the reporting year 2014 to examine factors associated with the closure of the obstetrics department by 2019. The variable *obstetrics department closed by 2019* served as dependent variable, whereas the variables *annual number of live births in hospitals*, *ownership*, *availability of a pediatrics department on site*, *academic teaching hospital*, *population density*, and *minimal travel time between two hospital sites with an obstetrics department* served as independent variables.

As a prerequisite for logistic regression, variables were checked for multicollinearity and linearity of logit. If multicollinearity existed in the final model, variables were removed. If variables showed non-normal distribution, median and interquartile ratios are reported. For all significance tests in the final regression model, we used an alpha level of 0.05. To address multiple testing, we chose to use the approach by Benjamin and Hochberg and control for the false discovery rate [35]. To demonstrate goodness of fit, we calculated McFadden´s Pseudo R^2^ and the Akaike information criterion (AIC) for each model. We used backward stepwise regression by AIC to choose the model with the best fit.

Concerning the annual number of live births, the literature suggests different cut off points at which the probability of obstetrics department closure increases dramatically. To investigate this graphically for Germany, we performed locally weighted scatterplot smoothing for this variable.

To assess the accessibility of hospital sites with an obstetrics department in Germany in 2014 and 2019, we used travel times to the nearest service. We depicted driving times from any place in Germany to the next hospital site with a department of obstetrics. A map was created with addresses from hospital sites with an obstetrics department provided in the quality reports and OpenStreetMap (leaflet package [2.0.4.1]). A model was built using a grid plotting 100,000 random points on the map of Germany. The minimum driving times from these random points to the closest hospital site with an obstetrics department were calculated. Driving times were determined via the open-source routing for shortest paths in road networks OSRM (osrm package [3.4.1]). As driving times over 30 min are considered critical for timely patient care [15, 29], these driving times were chosen to be visualized on the final map. Furthermore, driving times over 40 min, as recommended by the G-BA, were also highlighted [36]. In addition to the status quo scenario, we also depicted driving times for a scenario in which only hospital sites with an obstetrics department and a pediatrics department are considered and a scenario in which only hospitals with at least 600 live births are considered for 2019. All data analyses were performed using R Studio version 1.4.1106.

## Results

For the reporting year 2014: 747, for the year 2019: 662 hospital sites with an obstetrics department were identified (-12.6%, closure of 85 departments). Of these closures, 13 were identified as total hospital closures. In 2014, 41.23% of hospital sites with an obstetrics department also disposed of a pediatrics department. This proportion slightly increased in 2019 to 45.92%. The median number of annual live births at a hospital site increased from 702 in 2014 to 879 in 2019 (+ 25.21%). In both years, more than half of all hospital sites were situated in highly populated areas. In 2014, 19.01% of all hospital sites were located in areas with a high fertility rate; by 2019, this figure had risen to 53.10%. More detailed information on the characteristics of the analyzed hospital sites is displayed in Table 1.

**Table 1:** Characteristics of hospital sites with an obstetrics department (2014 and 2019)

	Hospital sites with an obstetrics department in 2014	Hospital sites with an obstetrics department in 2019	
Variable	N = 747	N = 662	p-value
**Live births**, Median (IQR^1^)	702 (453;1,181)	879 (577;1,511)	
*Live births, Mean (Min*^*2*^;*Max*^*3*^*)*	*910.5 (15;5,081)*	*1127.2 (32;5,670)*	**< 0.001** ^4^
**Ownership**, n (%)			0.445^5^
non-profit hospital	289 (38.69%)	244 (36.86%)	
public hospital	336 (44.98%)	301 (45.47%)	
private hospital	122 (16.33%)	117 (17.67%)	
**Pediatrics department**, n (%)			0.298^5^
No	439 (58.77%)	358 (54.08%)	
Yes	308 (41.23%)	304 (45.92%)	
**Academic teaching hospital**, n (%)			0.078^5^
No	245 (32.80%)	161 (24.32%)	
Yes	470 (62.92%)	460 (69.49%)	
University hospital	32 (4.28%)	41 (6.19%)	
**Population density**, n (%)			**0.041** ^5^
low population density	35 (4.69%)	20 (3.02%)	
medium population density	298 (39.89%)	241 (36.40%)	
high population density	414 (55.42%)	401 (60.57%)	
**Fertility rate**, n (%)			**0.002** ^5^
low fertility rate	42 (5.62%)	32 (4.84%)	
medium fertility rate	536 (75.37%)	278 (42.06%)	
high fertility rate	142 (19.01%)	351 (53.10%)	
**Minimal travel time between two hospital sites with an obstetrics department**^**6**^, Median (IQR)	18.2 (9.55,25.30)	18.80 (9.67,26.67)	
*Minimal travel time between two hospital sites with an obstetrics department*^*1*^, *Mean (Min*^*2*^;*Max*^*3*^*)*	18.1 (0.3;72.2)	19 (0.7;57.6)	0.1257^4^
**Obstetrics department closed by 2019**, n (%)			
No	662 (88.62%)	-	
Yes	85 (11.38%)	-	

^1^IQR: Interquartile range, ^2^Min: Minimum, ^3^Max: Maximum, ^4^Welch Two Sample t-test, ^5^Pearson’s Chi-squared test, ^6^Driving times in minutes

### Regression analysis

Because no obstetrics departments were closed in university hospitals, they were omitted from the analyses. Furthermore, cases for which the entire hospital closed down have been excluded from the analysis because other factors may have influenced the closure of the obstetrics department, leaving 702 hospital sites in the model. Table 2 shows the descriptive data for the regression sample.

**Table 2:** Characteristics of regression sample of hospital sites with the department of obstetrics (2014)

	Hospital sites with an obstetrics department in 2014
Variable	N = 702
*Dependent variable*	
**Obstetrics department closed by 2019**, n (%)	
No	630 (89.74%)
Yes	72 (10.26%)
*Independent variables*	
**Ownership**, n (%)	
non-profit hospital	280 (39.89%)
public hospital	303 (43.16%)
private hospital	119 (16.95%)
**Pediatrics department**, n (%)	
No	425 (60.54%)
Yes	277 (39.46%)
**Academic teaching hospital**, n (%)	
No	236 (33.62%)
Yes	466 (66.38%)
**Population density**, n (%)	
low population density	35 (4.99%)
medium population density	292 (41.60%)
high population density	375 (53.42%)
**Fertility rate**, n (%)	
low fertility rate	35 (4.99%)
medium fertility rate	527 (75.07%)
high fertility rate	140 (19.94%)
**Live births**, Median (IQR)	683 (450,1,126)
**Minimal travel time between two hospital sites with an obstetrics department**^**1**^, Median (IQR)	18.2 (9.55,25.30)
*Minimal travel time between two hospital sites with an obstetrics department*^*1*^, *Mean (Min*^*2*^;*Max*^*3*^*)*	18.6 (0.30;72.20)

^1^Driving times in minutes, ^2^Min: Minimum, ^3^Max: Maximum

Prior to the use of stepwise regression, we computed a full regression model including all variables. In this model the variables medium population density vs. low population density (p = 0.020), the annual number of live births (p < 0.001), and minimal travel time between two hospital sites with an obstetrics department (p = 0.030) are significantly associated with the closure of the obstetrics department after the correction of multiple testing. Table 3 shows the full regression model.

**Table 3:** Multivariate regression analysis of factors associated with the closure of obstetrics departments

Characteristic	OR^1^	95% CI^2^	GVIF^3^	p-value^4^
**Ownership**			1.1	
private hospital	—	—		
non-profit hospital	0.995	0.457, 2.225		> 0.9
public hospital	0.796	0.373, 1.748		0.6
**Pediatrics department**			1.1	
No	—	—		
Yes	0.373	0.131, 0.916		0.079
**Academic teaching hospital**			1.1	
No	—	—		
Yes	0.827	0.449, 1.517		0.6
**Population density**			1.2	
low population density	—	—		
medium population density	0.239	0.089, 0.651		0.020
high population density	0.253	0.076, 0.842		0.055
**Fertility rate**	0.158	0.012, 2.101	1.1	0.2
**Live births**	0.995	0.993, 0.996	1.2	< 0.001
**Minimal travel time between to hospital sites with an obstetrics department**	0.952	0.916, 0.987	1.3	0.030

^1^OR = Odds Ratio, ^2^CI = Confidence Interval, ^3^GVIF = Generalized Variance Inflation Factor, ^4^False discovery rate correction for multiple testing, Null deviance = 464; Null df = 701; Log-likelihood = -166; Akaike information criterion = 353; Bayesian information criterion = 398; Deviance = 333; Residual df = 692; McFadden’s adjusted R2: 0.240; Number of observations = 702

The deployment of backward stepwise regression yielded a final regression model including the variables *availability of a pediatrics department* on site, *population density*, *fertility rate*, annual *number of live births in a hospital site*, and minimal travel time between two hospital sites with an obstetrics department. The final regression model is displayed in Table 4.

**Table 4:** Backward stepwise regression analysis of factors associated with the closure of obstetrics departments

Characteristic	OR^1^	95% CI^2^	GVIF^3^	p-value^4^
**Pediatrics department**			1.0	
No	—	—		
Yes	0.357	0.126, 0.863		0.04
**Population density**			1.2	
low population density	—	—		
medium population density	0.24	0.09, 0.648		0.012
high population density	0.251	0.077, 0.822		0.033
**Fertility rate**	0.157	0.012, 2.046	1.1	0.2
**Live births in hospital**	0.995	0.993, 0.996	1.1	< 0.001
**Minimal travel time between two hospital sites with an obstetrics department**	0.95	0.915, 0.985	1.3	0.012

^1^OR = Odds Ratio, ^2^CI = Confidence Interval, ^3^GVIF = Generalized Variance Inflation Factor, ^4^False discovery rate correction for multiple testing, Null deviance = 464; Null df = 701; Log-likelihood = -167; Akaike information criterion = 348; Bayesian information criterion = 380; Deviance = 334; Residual df = 696; McFadden’s adjusted R2: 0.251; Number of observations. = 702

In the final model, all variables are significantly associated with the closure of the obstetrics department except fertility rate. The variable pediatrics departments shows a strong negative association with the dependent variable obstetrics department closed by 2019 (OR = 0.357; 95% CI = 0.126, 0.863). Thus, the odds of a hospital site with an obstetrics department and an additional pediatrics department to close down their obstetrics department are approximately three times lower compared to an obstetrics department without an additional pediatrics department. The same applies for the variable population density. For a hospital site to be located in an area with a medium population density (OR = 0.24, 95% CI = 0.09–0.648) or high population density (OR = 0.251, 95% CI = 0.077–0.822) the odds for the obstetrics department to close down are 4.2 (4 respectively) times lower compared to hospital sites that are located in areas with a low population density. Also, the variables annual number of livebirths and minimal travel time between two hospital sites with an obstetrics department showed a negative association with the dependent variable. For every additional child born (OR = 0.995, 95% CI = 0.993–0.996) and for every additional minute of driving time between hospital sites with an obstetrics department respectively (OR = 0.95, 95% CI = 0.915–0.985) the odds for the obstetrics department to close decrease. The final regression model showed a slightly better fit (R^2^: 0.251) compared to the initial regression model including all variables (R^2^: 0.24).

**Fig. 1:**
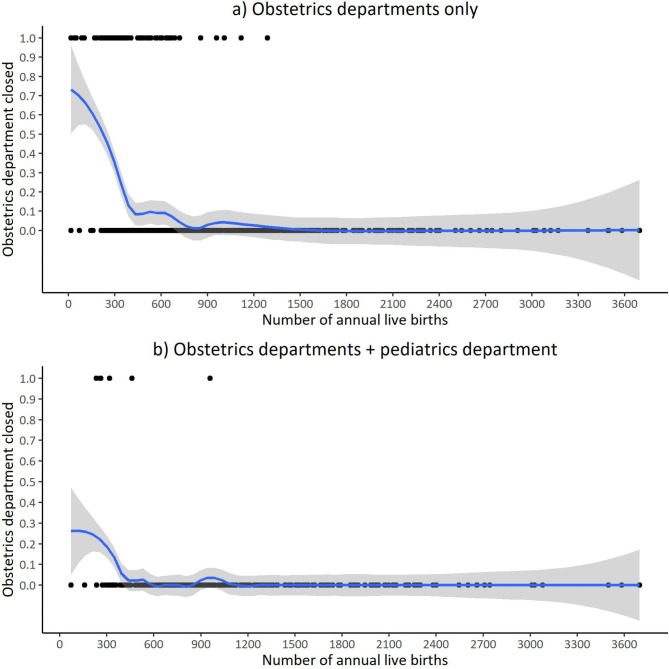
Locally weighted scatterplot smoothing of variables obstetrics department closed and live births

Figure 1 shows the detailed investigation of the variable annual number of live births using locally weighted scatterplot smoothing. We performed two analysis: one for obstetric departments only and one for obstetrics department with an additional pediatrics department. From the data in Fig. 1, it is apparent that first obstetrics departments with an additional pediatrics department face a lower probability of closure compared to hospital sites with an obstetrics department only. Second, above the threshold of annual live births between the 25th and 50th percentile (450 and 683) the probability for obstetrics department closure decreases crucially. A small ascent of the probability of obstetrics department closure can be observed between 900 and 1200 livebirths for both, hospitals with an obstetrics department only and hospital sites with an additional pediatrics department.

### Accessibility of hospital sites with an obstetrics department

**Fig. 2:**
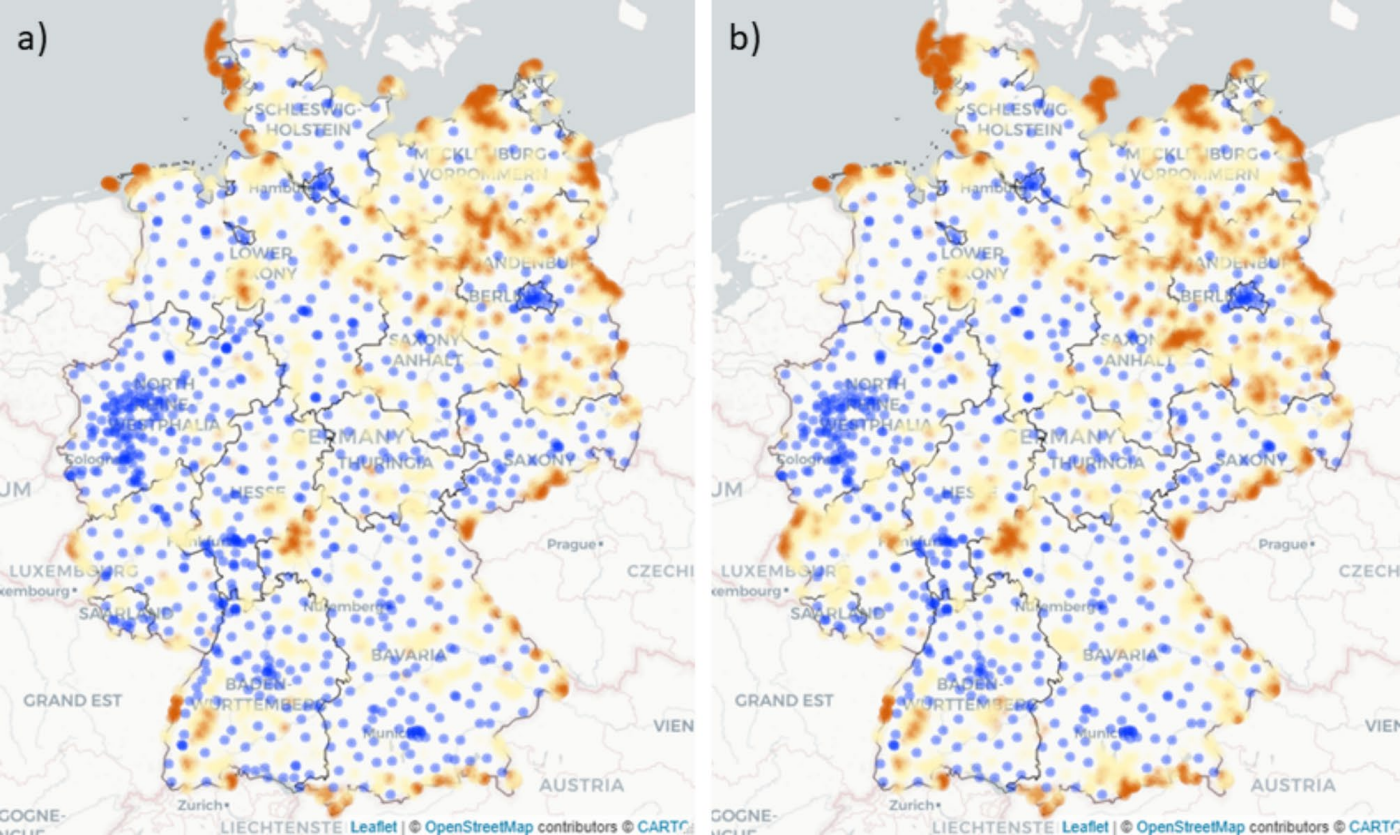
Driving times to the next hospital site with an obstetrics department in 2014 and 2019 Driving times to (a) all hospital sites with an obstetrics department in 2014 (b) hospital sites with an obstetrics department in 2019; marked in yellow: driving times between 30 and 40 min: marked in orange driving times over 40

**Fig. 3:**
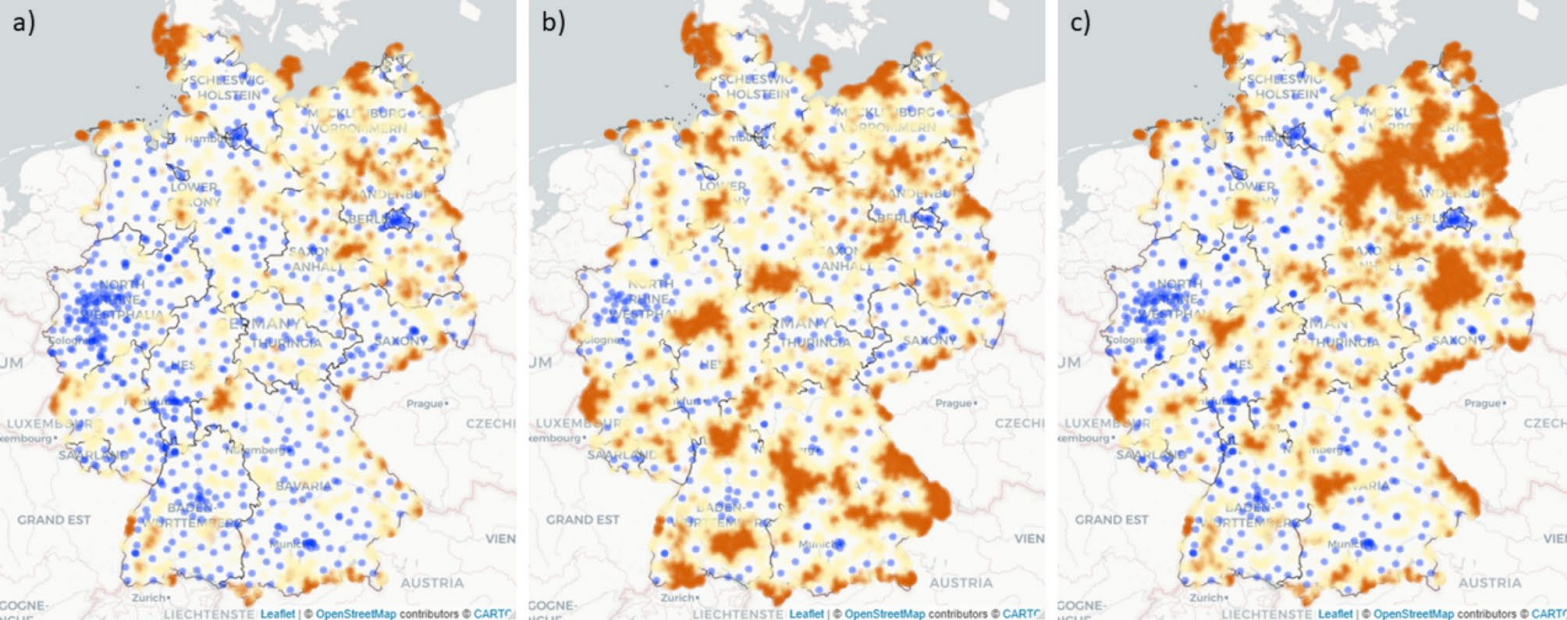
Driving times to the next hospital site with an obstetrics department in 2019 compared to driving times resulting from different regionalization scenarios Driving times to (a) all hospital sites with an obstetrics department in 2019 (b) all hospital sites with an obstetrics department and an additional pediatrics department in 2019 (c) all hospital sites with an obstetrics department and ≥ 600 live births in 2019; marked in yellow: driving times between 30 and 40 min: marked in orange driving times over 40

Figure 2 highlights areas in Germany in which driving times to the next hospital site with an obstetrics department exceeded the 30 or 40 min threshold in 2014 (Fig. 2a) and 2019 (Fig. 2b). Additionally, Fig. 2 displays all hospital sites with an obstetrics department in 2014 (Fig. 2a) and 2019 (Fig. 2b). Areas in which driving times exceed the 30 min threshold are highlighted in yellow. Areas in which driving times exceed the 40 min threshold are highlighted in orange. The map in Fig. 2 demonstrates that for most areas in Germany, hospital sites with an obstetrics department were reachable by car in under 30 min. Areas where this is not the case are mostly located in the north east of Germany. In addition, because hospitals with an obstetrics department do not exist on most of the German islands in the north west, driving times exceeded the 40 min threshold in these areas. When comparing driving times in 2014 with driving times in 2019 (Fig. 2a and b), the areas exceeding a driving time of 30 min by car have slightly increased in 2019. Figure 3 shows driving times resulting from different regionalization scenarios. To enable a comparison with the actual situation in 2019, Fig. 3a shows driving times to all hospital sites with an obstetrics department in 2019. To address the G-BA’s resolution that a high standard of care (i.e., availability of pediatric care) justifies driving times of 40 min, Fig. 3b displays driving times for hospital sites with an obstetrics department and a pediatrics department in 2019. As the total number of hospital sites decreases when considering only hospital sites with an obstetrics department and a pediatrics department (Fig. 3b), areas in which driving times exceed the 30 or 40 min threshold increase. Specifically, areas in which driving times exceed the 40 min threshold result from this scenario. Figure 3c presents a scenario of driving times to hospital sites with an obstetrics department and at least 600 live births in 2019. Particularly in the north east of Germany, driving times increase in this scenario. Dynamic maps for the accessibility analysis can be found via the link provided in the data availability section.

## Discussion

The aims of the present study were twofold: to assess factors associated with the closure of an obstetrics department in German hospitals and to visualize the impact of obstetrics department closure on accessibility.

Regarding the first aim, our study findings suggest that a higher annual number of live births, the availability of a pediatrics department, the hospital being located in either medium or high population areas, and longer travel times between two hospital sites with an obstetrics department decrease the likelihood of obstetrics department closure. Ownership, fertility rate, and the hospital site being an academic teaching facility were not significantly associated with obstetrics department closure.

Our findings align with the findings of Albrecht et al., who stated that hospital sites with lower annual number of livebirths are especially likely to close their department of obstetrics and gynecology [13]. Also, Mennicken et al. suggest that for hospitals to be able to maintain economic viability, obstetrics departments need to have a minimum number of cases [21]. With regard to the closure of obstetric departments, our analyses show a risk reduction starting at 600 births per year. Previous findings suggest that for Germany to guarantee continuous midwifery care (when factors such as the availability of a neonatal unit or a 24 h laboratory are not considered), which is mandatory in Germany, hospitals with an obstetrics department would reach full utilization at a rate of at least 600 annual live births [13]. The small increase in the probability for closure between 900 and 1,200 livebirths can be explained with the sensibility of the analysis used. In these birth range only 1 out of 308 (0.32%) hospitals with an obstetrics department and an additional pediatrics department and 2 out of 439 (0.46%) hospitals with an obstetrics department and no additional pediatrics department closed. The fact that shorter travel times between two hospital sites with an obstetrics department are associated with obstetrics department closure may have several reasons. First, hospital sites that are located in the same catchment area compete for the same patients and staff. This may eventually lead to obstetrics department closure in these areas. Our results show, that the absolute number of obstetrics department closures between 2014 and 2019 was higher in areas with a medium or high population density combined than in areas with a low population density. Previous reports support our findings that the absolute number of obstetrics department closures is higher in high populated areas compared to low populated areas [13, 37]. Second, hospital sites with long distances to the next hospital site may not be able to close down their obstetrics department because of the securement of healthcare provision in that area. However, the effect of closure of an obstetrics department on accessibility and driving times is lower in areas with a high density of hospitals with an obstetrics department than in areas with a low density of hospitals with an obstetrics department. Controversially, our model suggests that hospitals in areas with a medium or high population density have lower odds to close their obstetrics department compared to obstetrics department in areas with a low population density. The association of the availability of a pediatrics department with obstetrics department closure indicates that medical services are consolidated in hospital sites where specialized staff is available.

Regarding the accessibility of obstetrics departments, we conclude that for most areas in Germany, driving times of less than 30 min to the next hospital site with an obstetrics department could be guaranteed in 2014 and 2019. In general, it must be noted that longer travel times are observed primarily in rural regions. We showed that further regionalization (when only hospitals with an obstetrics department and a pediatrics department remain open; when only hospitals with at least 600 live births remain open) will have an impact on accessibility and driving times over the threshold of 30 or 40 min increased for large areas. For these areas timely access to care cannot be guaranteed if the spatial distribution of hospital sites remains unchanged. There are different opinions on driving time thresholds to obstetric facilities. The most common used threshold for obstetric services in countries such as Germany or Japan is a 30 min driving time by car [15, 21]. There are only few studies on the impact of travel times and complications in childbirth. For example, Ravelli and colleagues demonstrated that a driving time of 20 min or more was associated with an increased risk of mortality and adverse outcomes in woman at term in the Netherlands [38]. However, the authors state that the 20 min threshold was based on travel under the best conditions, assuming that real travel times were probably longer and closer to the 30 min threshold.

Koike and colleagues concluded that regionalization of obstetric services impairs access [15]. Overall, our analyses agree with these findings and give answers on how specific regionalization scenarios would impact accessibility. However, the question remains who is impacted by impaired accessibility. In Portugal, in the context of regionalization, national policies demanded obstetrics department closure for hospitals with less than 1,500 births annually, resulting in the closure of more than 150 maternity units. In-hospital births increased from 74% to 99% and neonatal mortality decreased significantly from 8.1 to 1,000 livebirths to 2.7 per 1,000 livebirths [39]. Longer travel times can be a burden to parents as out of pocket payments for gas, hotel or child care increase [6] and may lead to health care inequality. Also, emergencies where timely access to care is crucial suffer from longer travel times.

There is a trade-off between accessibility and the positive effects of regionalization (i.e., improved patient outcomes, better staffing, and reduced costs [15]). If the goal is to ensure care close to home, it is important to consider how high-quality care can be provided under these conditions. To guarantee both accessibility and expertise, obstetric hubs need to be spatially equally distributed. Survey data from a large German health insurance company showed that, in 2013, 60% of pregnant woman chose the closest hospital to give birth [40]. Only 14% of pregnant woman were willing to drive twice as long to choose an appropriate hospital [40]. Some countermeasures to avoid undersupply in the field of obstetrics already exist in Germany. For instance, the government offers special boarding programs, in which pregnant woman who live on the German islands receive paid housing on the mainland 2 weeks prior to the calculated date of birth [41]. In addition, some federal states increased delivery room capacities and apprenticeship capacities for midwives to counteract staff shortages [41].

### Strengths and limitations

In this study, we used a data source that enabled us to provide a complete picture of all German hospital sites with an obstetrics department in 2014 and 2019. In addition to the descriptive data presentation, we performed a multivariate regression analysis to identify factors associated with the closure of hospital sites with an obstetrics department. We further managed to model the shortest driving times to the next hospital site with an obstetrics department on the basis of actual travel times. However, results within this study have to be regarded with caution owing to the following limitations: The primary data source for this study included quality reports of German hospital sites. Although the completeness of this data source keeps improving with time, it was noted during data analysis that not all information in the dataset was complete. However, for the purpose of the research question addressed in this study, this data source is the best source currently available, and data were checked for plausibility. We performed our spatial analyses from a public health perspective. We recognize that, from a transport geography perspective, more advanced spatial analyses can be useful [42]. In Germany, deliveries take place not only in clinics but also in birth centers under the supervision of midwives. We did not include these centers in our accessibility model, as these are not part of inpatient care. As already pointed out in the background section, only a small proportion of all births in Germany are out-of-hospital births. Apart from accessibility and spatial distribution of hospital sites, quality of care and cost is a key criterion, which this study could not examine in detail considering the primary data source. These analyses are important to determine if the impact of regionalization in obstetrics on accessibility impacts health outcomes and if yes, in what way.

## Conclusion

Regionalization of obstetrics yields improved patient outcomes, more efficient staffing in times of labor shortage, and reduced costs owing to savings on equipment. Simultaneously, running fewer hospital sites with an obstetrics department impairs regional accessibility. Thus, policy makers encounter a challenge with weighing both aspects. Higher case numbers (number of live births), longer distances between hospitals sites with an obstetrics department, and the availability of a pediatrics department decrease the likelihood of obstetrics department closure. Currently, accessibility to hospital sites with an obstetrics department in Germany remains good, albeit with regional differences. Further regionalization of obstetric care will impact accessibility if obstetric hubs are not spatially equally distributed.

## Data Availability

The datasets generated and analyzed during the current study are available from the corresponding author on reasonable request. The structured quality reports of all acute hospital sites in Germany can be freely accessed under http://www.g-ba.de. More detailed dynamic maps for the accessibility analysis can be accessed via https://janhoffmann.shinyapps.io/Obstetrics_in_Germany/ Detailed information on the dynamic maps can be accessed via https://github.com/Jan-Hoffmann-21/ObstetricsinGermany.

## Abbreviations

G-BA: Federal Joint Committee
AIC: Akaike information criterion
